# Kinetics of the Gas-Phase Reaction of Hydroxyl Radicals with Trimethyl Phosphite (TMPi) over the 295–735 K Temperature Range

**DOI:** 10.1021/acs.jpca.6c02720

**Published:** 2026-08-14

**Authors:** Xiaokai Zhang, Pavel V. Koshlyakov, Evgeni N. Chesnokov, Lev N. Krasnoperov

**Affiliations:** † Department of Chemistry and Environmental Science, 5965 New Jersey Institute of Technology, Newark, New Jersey 07102, United States; ‡ Institute of Chemical Kinetics and Combustion, 104681 Siberian Branch of Russian Academy of Sciences, Novosibirsk 630090, Russian Federation

## Abstract

The kinetics of the gas-phase reaction between hydroxyl radicals (OH) and trimethyl phosphite (TMPi, (CH_3_O)_3_P): (OH + TMPi → products (reaction 1) was investigated over the 295–735 K temperature range at a total pressure of 1 bar using helium as the bath gas. Hydroxyl radicals were generated *in situ* via the rapid reaction of electronically excited oxygen atoms, O­(^1^D), with water vapor, providing a clean source of OH radicals. The time-resolved OH concentration profiles were monitored by ultraviolet absorption near 308 nm using a DC-discharge H_2_O/Ar low-pressure resonance lamp. The pseudo-first-order rate coefficients were obtained from exponential fits to the initial portions of the OH temporal profiles and converted to the bimolecular rate constants based on the TMPi concentrations. The reaction rate constant shows a pronounced negative temperature dependence across the studied temperature interval, with the reaction rate decreasing as the temperature increases. This behavior is consistent with that observed for related organophosphorus esters, including trimethyl phosphate and dimethyl methyl phosphonate, and supports a mechanistic interpretation involving a submerged transition state along the reaction coordinate. The measured rate constant is represented by the Arrhenius expression over the 295–735 K range: *k*_1_ = 1.27 × 10^–11^exp­(619 K/T) cm^3^ molecule^–1^ s^–1^, corresponding to an apparent negative activation energy of −5.15 kJ mol^–1^ (−1.23 kcal mol^–1^). The magnitude and the negative temperature dependence of *k*_1_ suggest that attractive long-range interactions and barrierless or near-barrierless channels dominate the entrance dynamics. These results extend the kinetic database for OH reactions with reduced phosphorus compounds and provide constraints for detailed reaction mechanisms relevant to phosphorus-containing species in combustion and atmospheric oxidation environments.

## Graphical abstract

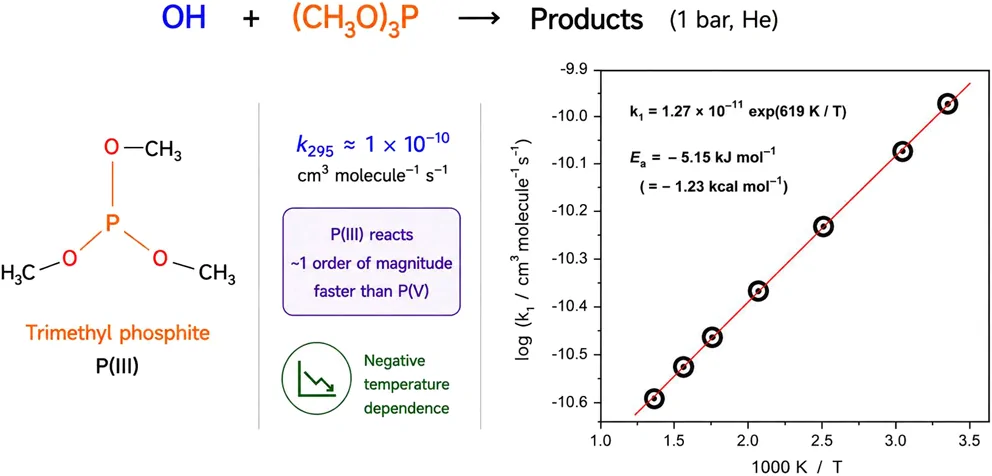

## Introduction

Organophosphorus flame retardants (OPFRs) constitute an important class of phosphorus-containing additives that are widely incorporated into commercial and industrial materials to reduce flammability and inhibit flame propagation.^1^ They are commonly incorporated into polymers, electronic equipment, housings, textiles, foams, and furniture coatings, where they act primarily through the gas-phase and condensed-phase chemical mechanisms.^2^ In the gas phase, OPFRs interfere with combustion chain reactions by scavenging key oxidizing radicals, most notably hydroxyl (OH), which are the major chain carriers in hydrocarbon oxidation.^3^ Phosphorus-containing intermediates and decomposition products formed during thermal degradation can react rapidly with these radicals, thereby shortening radical chain lengths and lowering overall heat release rates.^4^ Consequently, flame speed and flame stability decrease, leading to enhanced fire resistance.

OPFRs have increasingly replaced legacy halogenated flame retardants, such as polybrominated diphenyl ethers (PBDEs) and certain chlorinated additives, in response to regulatory restrictions and concerns about persistence, bioaccumulation, and toxicity of halogenated species.^5–7^ Phosphorus-based alternatives are often considered more environmentally acceptable and, in many formulations, comparably or more effective at lowering flammability.^8^ Nevertheless, increasing evidence indicates that some OPFRs and their degradation products may pose health and environmental risks, including developmental and neurotoxic effects, endocrine disruption, and long-term environmental persistence.^6,9^ These concerns have motivated renewed interest in understanding their detailed thermal decomposition and oxidation chemistry, particularly under combustion-relevant conditions.

From a mechanistic perspective, quantitative kinetic data for elementary reactions between OPFRs and key combustion radicals are required to construct predictive oxidation and inhibition models. Among these radicals, OH plays a dominant role because it is typically the most abundant and reactive oxidizing species in flames and high-temperature oxidation environments.^10^ Accurate bimolecular rate constants and temperature dependences for OH reactions with organophosphorus compounds are therefore critical inputs for chemical kinetic mechanisms used in combustion simulations and fire suppression modeling.

Previous work from this laboratory examined the gas-phase kinetics of OH reactions with two representative OPFR molecules, trimethyl phosphate (TMP) and dimethyl methylphosphonate (DMMP).^11,12^ In both systems, the measured rate constants displayed a characteristic non-Arrhenius, “V”-shaped temperature dependence, consistent with a submerged transition state on the potential energy surface.^13^ Such behavior suggests that attractive entrance-channel interactions play a role in determining reactivity for this class of compounds. To further probe the generality of this mechanism and to assess the influence of phosphorus oxidation state on OH reactivity, trimethyl phosphite (TMPi), (CH_3_O)_3_P, was selected for the present study. TMPi contains phosphorus in a lower oxidation state than TMP and DMMP, enabling systematic comparison across closely related molecular structures.

The target reaction investigated here is

1 $$\mathrm{OH}+\mathrm{TMPi}\left({\left({\mathrm{CH}}_{3}O\right)}_{3}P\right)\to \mathrm{products}$$

At present, only a single experimental measurement of the rate constant of this reaction at ambient temperature using a relative rate technique has been reported, *k*_1_ = 7.1 × 10^–10^ cm^3^ molecule^–1^ s^–1^.^14^ This value exceeds the expected hard-sphere collision limit, raising questions about possible secondary chemistry, reference reaction uncertainties, or analytical artifacts in the measurements. Independent absolute-rate measurements over a broad temperature interval are therefore desirable to resolve the issue and to establish more reliable kinetic parameters.

In the present work, the kinetics of reaction 1 was studied using a direct technique of pulsed laser photolysis–transient UV absorption (PLP–UV). Hydroxyl radicals were generated in a rapid reaction of electronically excited oxygen atoms O­(^1^D) with water vapor

2 O(D1)+H2O→2OH

Photolysis of N_2_O at 193 nm (ArF excimer laser) was used to generate electronically excited atoms O­(^1^D)

3 N2O+hv(193nm)→O(D1)+N2

Time-resolved OH concentration profiles were monitored via UV absorption near 308 nm following the laser pulse, allowing direct determination of OH temporal decays in the presence of TMPi. Experiments were conducted in a temperature-controlled flow reactor that enabled control of total pressure and temperature across extended ranges. The experimental configuration provides absolute rate coefficients, minimizes secondary chemistry, and permits detailed characterization of the temperature dependence and mechanistic features of OH + OPFR reactions.

## Experimental Section

Both experimental set-ups used at NJIT and the ICKC have been described in detail previously.^15–18^ Aside from several minor differences, the experimental setups at IChK&C and NJIT were nearly identical. In both cases, pulsed laser photolysis coupled with time-resolved UV transient absorption spectroscopy was utilized. The setup at the ICKC in this study was additionally equipped with a Fourier-Transform IR Spectrometer (Bruker Vector 22) downstream of the reaction vessel to assess the temperature stability range of the reactants mixture and possible dark reactions between the reactants. Another minor difference was in the material of the connection tubing through which the mixed reactants were transferred to the reactor (1/8” SS tubing at NJIT and 1/4” Teflon tubing at the ICKC).

The experiments were carried out in a temperature-controlled, high-pressure flow reactor using a pulsed laser photolysis–transient UV absorption (PLP–UV) technique. The overall configuration and reactor design have been described in detail earlier; only the key operational parameters and procedures relevant to the present study are summarized here.

Hydroxyl radicals were generated in situ by pulsed photolysis of N_2_O at 193.3 nm using an ArF excimer laser, followed by rapid reaction of the resulting electronically excited oxygen atoms O­(^1^D) with water vapor. The primary photochemical and radical-generation steps are N_2_O + *h*ν (193 nm) → O­(^1^D) + N_2_, O­(^1^D) + H_2_O → 2 OH, with the latter serving as the dominant channel under the experimental conditions.

The relative importance of the N_2_O photolysis channels and the subsequent O­(^1^D) reactions have been analyzed previously and is not repeated here.^18,19^ Experimental conditions were selected to ensure efficient conversion of O­(^1^D) to OH and to minimize secondary radical chemistry.

Before entering the reactor, the excimer laser beam was conditioned with a two-lens beam-shaping assembly to improve spatial uniformity in the photolysis zone. The measured fluence across the reactor cross section was uniform within ± 7.3% of the mean, ensuring nearly homogeneous radical production in the probed volume and reducing systematic errors in the derived kinetic traces. Time-resolved OH concentration profiles were measured by UV absorption near 308 nm using a multiline, low-pressure H_2_O/Ar DC discharge resonance lamp as the probe source.

Gas flows were metered using calibrated mass flow controllers (Brooks, model 5850). Helium was used as the bath gas and carrier. The reagents and the bath gas were flowing to the reaction cell via a 1/8” SS tubing. The total reactant flow entering the reactor was typically 11–12 sccs (in the gas equivalent units), with additional helium flush flows of 2 sccs directed across the reactor windows to prevent condensation and surface reactions. Liquid water and trimethyl phosphite (TMPi) were introduced using two precision syringe pumps (Harvard Apparatus, model PHD 4400). Each liquid was delivered through a capillary injector into a heated evaporator maintained at 90 °C to ensure complete vaporization prior to mixing with the carrier gas and entering the reactor.

The effective gas-phase flow rates of water and TMPi (expressed in sccm equivalents) were calculated from the measured liquid volumetric injection rates using the ideal gas law and known liquid densities and molar masses. Under the experimental conditions, the reactant concentrations were in the ranges (1.2–2.5) × 10^17^ molecules cm^–3^ for H_2_O, (6.8–13.7) × 10^16^ molecules cm^–3^ for N_2_O, and (0–3.7) × 10^14^ molecules cm^–3^ for TMPi. These concentration regimes ensured fast conversion of O­(^1^D) to OH radicals and the pseudo–first-order conditions with TMPi in excess relative to OH for kinetic analysis.

The optical absorption path length was 10 cm. Under the experimental conditions, the initial OH concentration was sufficiently low that the estimated pseudo-first-order loss due to OH self-reaction was less than 1% of the loss due to reaction with TMPi. Therefore, OH self-reaction was neglected in the kinetic analysis.

### Reagents

Helium used in the experiments was BIP Helium from Airgas with 99.9999% purity with reduced oxygen content (<10 ppb). N_2_O used in the experiments was from Airgas with 99.9999% purity. Purified water (Milli-Q) with TOC less than 5 ppb was degassed by freeze–pump–thaw cycles and used as a reactant supplied by a syringe pump (Harvard Apparatus PHD 4400) as well as in the low-pressure H_2_O/Ar discharge hydroxyl monitoring lamp. UHP Argon obtained from Matheson TriGas (99.999% purity) was used in the H_2_O/Ar lamp. TMPi was purchased from Aldrich, purity >98%.

## Results

The thermal stability of TMPi and the possibility of undesirable reactions among precursors and reactants were evaluated by IR spectroscopy (Bruker Vector 22) downstream of the reactor cell. The IR spectrum of a TMPi/He mixture at 1 bar and room temperature is shown in Figure 1. A sharp decrease in the TMPi IR absorption lines was observed above 748 K (Figure 2), indicating thermal decomposition of TMPi. The apparent activation energy of 43.7 kcal mol^–1^ (Figure 3) is comparable to the energy scale of C–H activation in TMPi.^20^ However, the relatively small pre-exponential factor (*A* ≈ 3.5 × 10^12^ s^–1^) is not typical of direct unimolecular C–H bond fission through a loose transition state. Therefore, the observed TMPi loss is attributed more generally to an apparent thermal decomposition process, which may include heterogeneous contributions.

**Figure 1:**
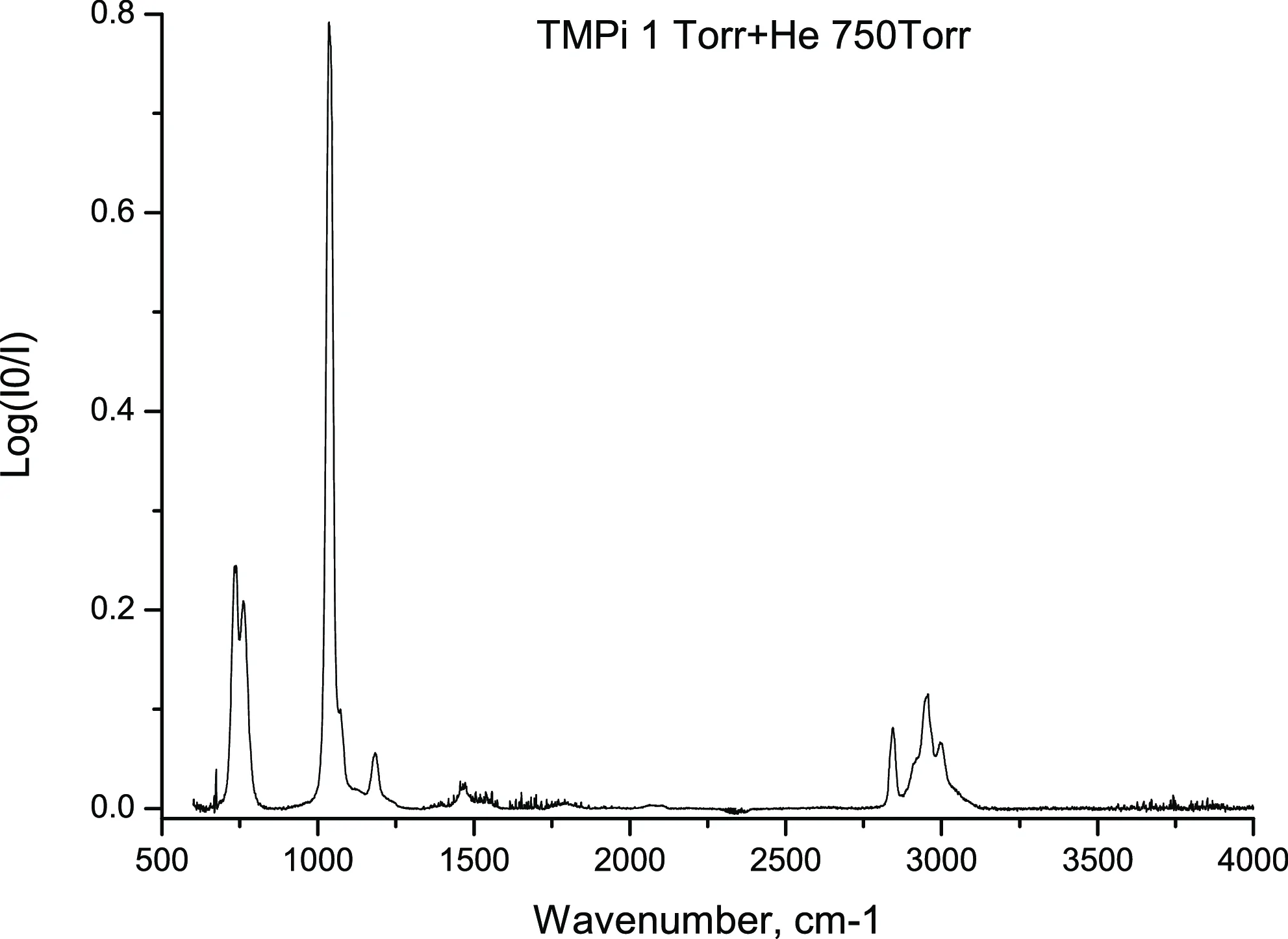
IR spectrum of TMPi recorded in a 17.7 cm gas cell containing 1 Torr TMPi in 750 Torr He at room temperature. The absorption lines at 1036.6 and 732 cm^–1^ were used in the measurements. The spectrum was recorded at a resolution of 0.5 cm^–1^.

**Figure 2:**
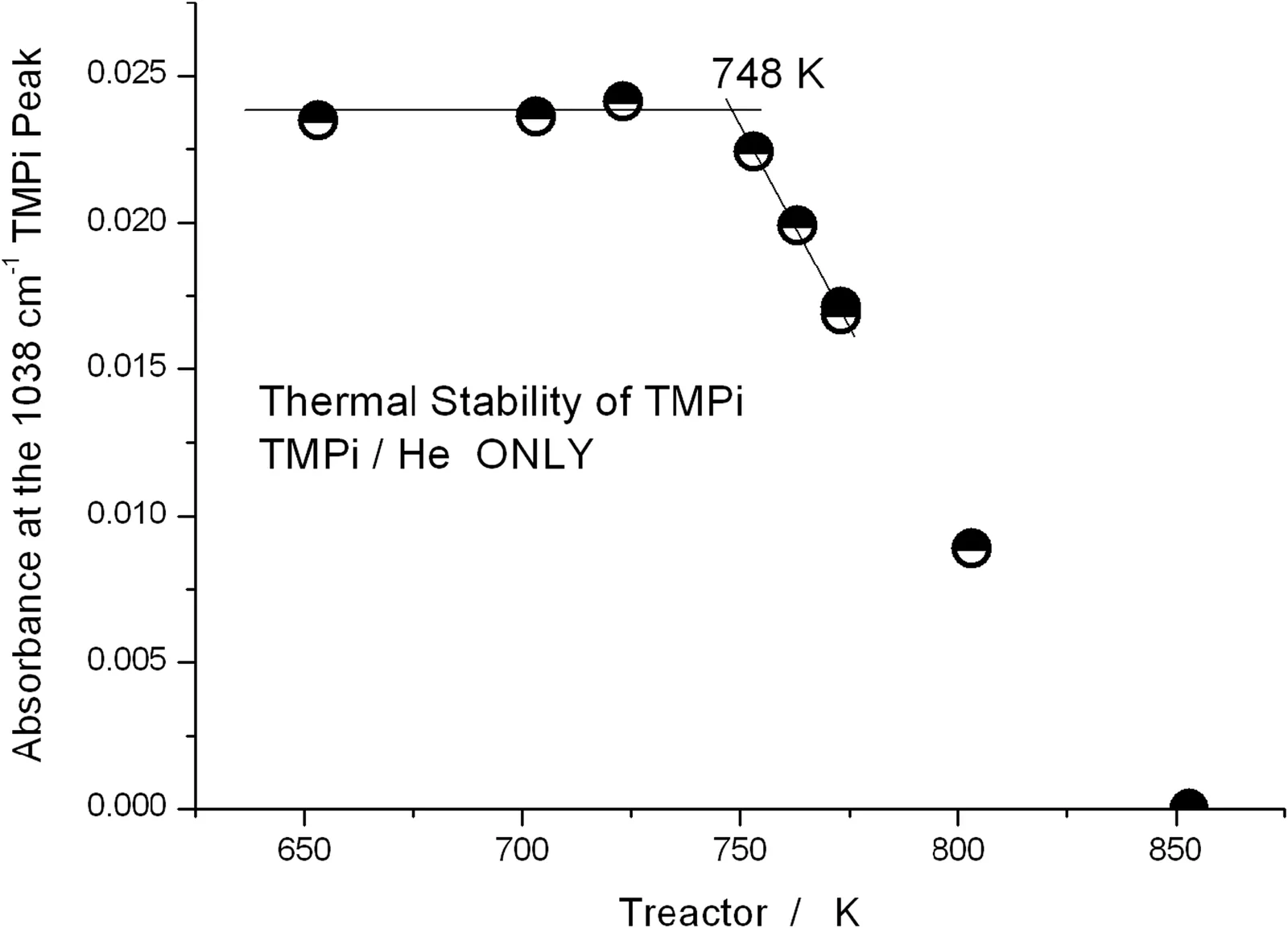
Thermal stability of TMPi measured by IR spectroscopy. The IR peak intensity of a TMPi/He mixture remains constant up to 735 K. Above 748 K, a sharp decrease in the IR line intensity is observed, indicating thermal decomposition of TMPi.

**Figure 3:**
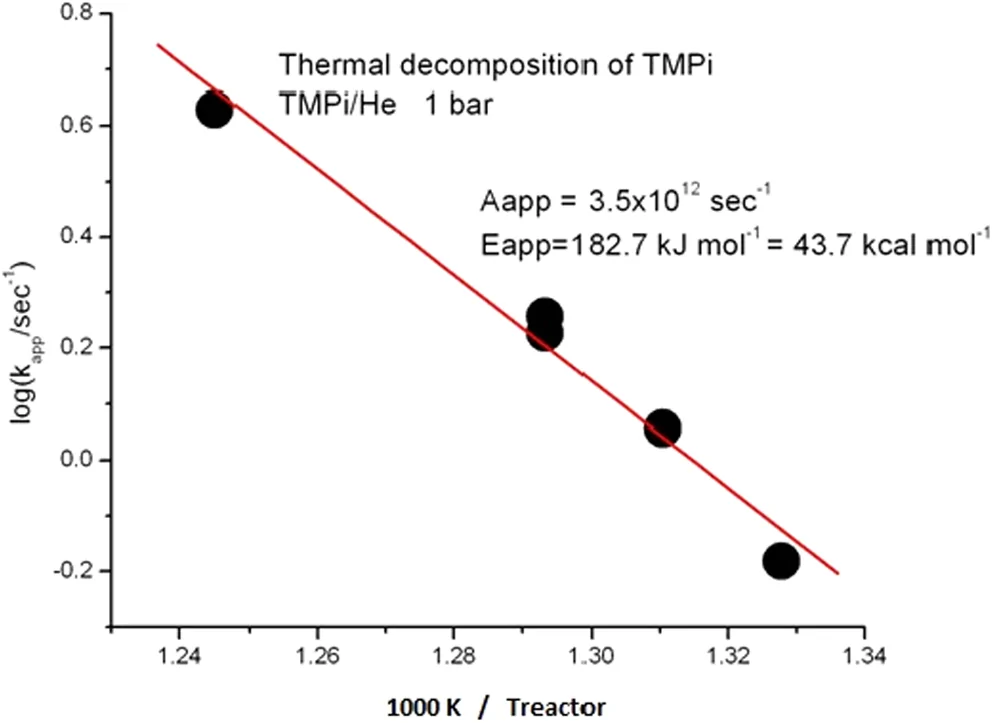
Arrhenius plot showing the apparent activation energy for TMPi pyrolysis at *T* > 735 K in 1 bar He.

As expected,^21–23^ TMPi reacted with water during transfer of the TMPi/H_2_O mixture to the IR observation cell. The products were dimethyl hydrogen phosphite ((CH_3_O)_2_HPO, DMHP) and methanol. Several observations suggested that this reaction was heterogeneous: its extent depended strongly on the flow history and the condition of the connecting tubing (fresh versus used), and the time required to reach a steady signal was much longer than the flush-out time of the tubing and IR cell.

Most kinetic measurements were performed at NJIT, where 1/8 in. OD stainless-steel tubing was used to transfer the reactant mixtures to the photolysis cell. Control experiments were also carried out in a configuration in which TMPi was mixed with the rest of the flow immediately before entering the photolysis cell.

Representative OH absorption profiles obtained for N_2_O/H_2_O/TMPi/He mixtures following 193 nm photolysis are shown in Figure 4. The rise in absorption corresponds to OH formation via reaction 2. Because the reaction of O­(^1^D) atoms with H_2_O is extremely fast (*k* = 1.8 × 10^–10^ cm^3^ s^–1^), the conversion time of excited oxygen atoms did not exceed 1 μs.

**Figure 4:**
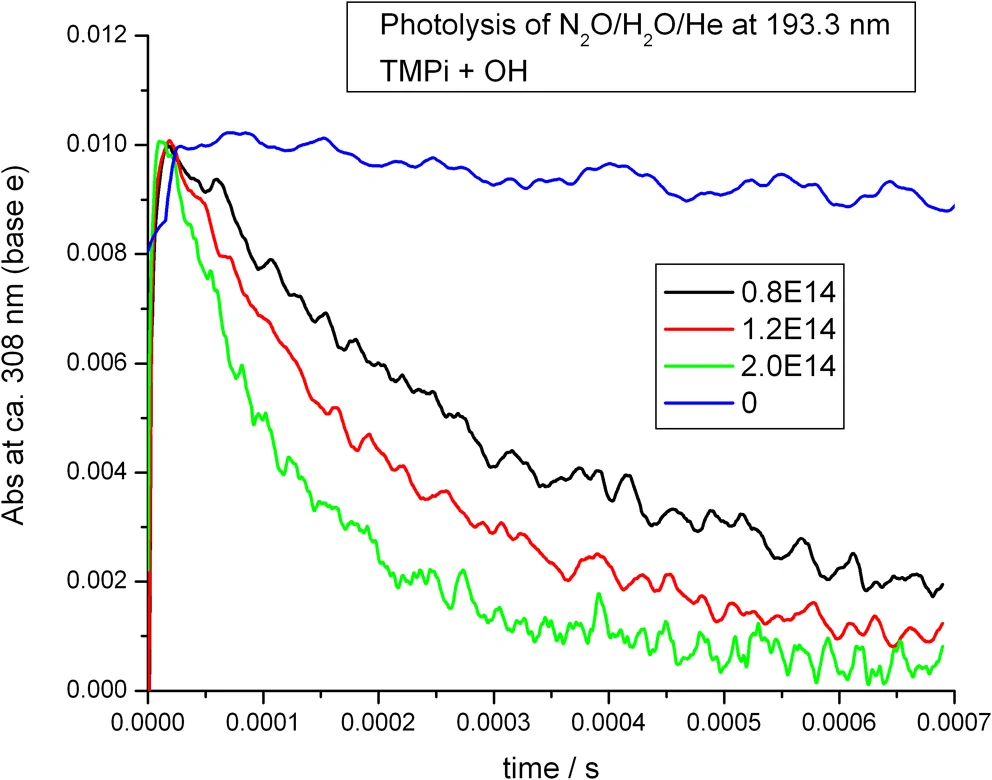
Representative OH UV absorption profiles near 308 nm following 193.3 nm photolysis of N_2_O/H_2_O/TMPi/He mixtures at *T* = 591 K and *p* = 1 bar. Blue, [TMPi] = 0; black, [TMPi] = 0.8 × 10^14^; red, [TMPi] = 1.2 × 10^14^; and green, [TMPi] = 2.0 × 10^14^ molecules cm^–3^.

A blank experiment performed in the absence of TMPi is included in Figure 4 as the reference OH temporal profile. Although TMPi absorbs 193 nm radiation and may undergo photodissociation, the observed linear dependence of the pseudo-first-order rate constants on TMPi concentration indicates that such secondary processes do not make a measurable contribution to the OH decay under the present experimental conditions.

The decay of the OH concentration reflects several processes, including OH self-reaction, wall loss, and the target reaction with TMPi. Secondary reactions of the products of reaction 1 may also contribute. In contrast to our previous studies of elementary reactions involving OH and other radicals, in which a full reaction mechanism was modeled by numerically solving the corresponding differential equations and fitting the calculated profiles to experiment, a simplified analysis was used here. For reaction 1, no information is currently available on the products, branching ratios, or rate constants of subsequent product reactions, making the previously used rigorous approach impractical. In the absence of a detailed reaction mechanism, integrated kinetic decay curves could not be derived. Therefore, the initial-rate method, which is widely used in chemical kinetics, was applied as described below.

Because TMPi was present in large excess over OH under pseudo-first-order conditions, its concentration remained effectively constant during the measurement. At early reaction times, the concentrations of products and secondary radicals are negligible, so the observed OH decay is dominated by reaction with TMPi. Restricting the fit to the initial one-third of the decay therefore minimizes the influence of secondary chemistry while maintaining a high signal-to-noise ratio.

After OH radicals are formed by reaction of excited oxygen atoms with water molecules, they are consumed by the target reaction 1 and by the side reactions discussed above. At short times, the overall OH loss rate constant, *k*′, can therefore be written as follows

E1a $$k^{\prime }={\left(-\frac{1}{{\left[\mathrm{OH}\right]}_{0}}\frac{d\left[\mathrm{OH}\right]}{dt}\right)}_{t=0}$$

E1b $$k^{\prime }={k_{1}}^{\prime }+k_{0}$$

E1c $${k_{1}}^{\prime }=k_{1}\left[\mathrm{TMPi}\right]$$

Here, *k*′ is the “initial-slope rate constant” defined by eq EE1a, *k*_1_′ = *k*_1_[TMPi] is the pseudo-first-order rate constant for the target reaction, and *k*_0_ accounts for all other loss processes. Exponential decay was used to evaluate the initial slopes because it provides an unbiased estimate of the initial decay rate for pseudo-first-order processes.

To determine the initial-slope rate constant, the initial portion of each decay profile (approximately the first third of the amplitude) was fitted with the exponential function

E2 $$\left[\mathrm{OH}\right]={\left[\mathrm{OH}\right]}_{0}\;\exp\left(-k’t\right)$$

Plotting the initial-slope rate constant, *k*′, against the reactant concentration [TMPi] is expected to yield a straight line. Its slope gives the rate constant for the target reaction 1, and its intercept gives the contribution of all other processes, such as self-reaction, at the initial stage.

The slope of the linear fit obtained at each temperature gives the rate constant under those conditions. Representative linear plots are shown in Figure 5.

**Figure 5:**
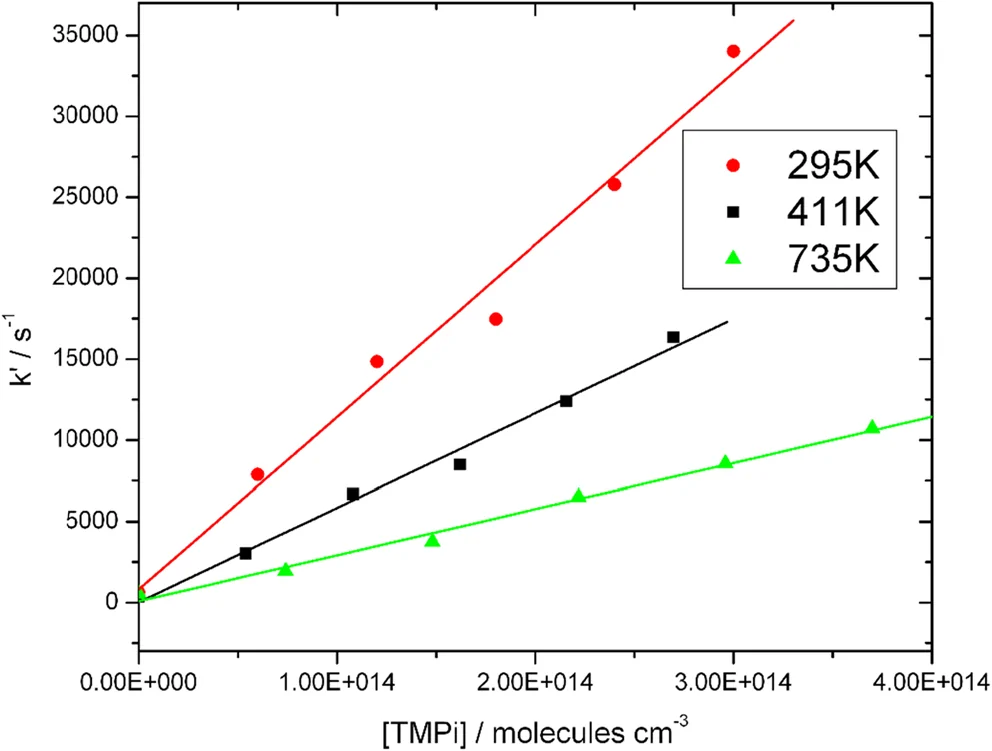
Dependence of the initial-slope rate constant, *k*′, on TMPi concentration at three temperatures. The mixtures were photolyzed at 193 nm. The slopes give the rate constant for reaction 1. The negative temperature dependence is evident. Red, 295 K; black, 411 K; and green, 735 K.

The results of all measurements are listed in Table 1. A clear negative temperature dependence is observed over the studied range. The measured rate constants were plotted against 1000/T in Arrhenius coordinates, as shown in Figure 6.

**Figure 6:**
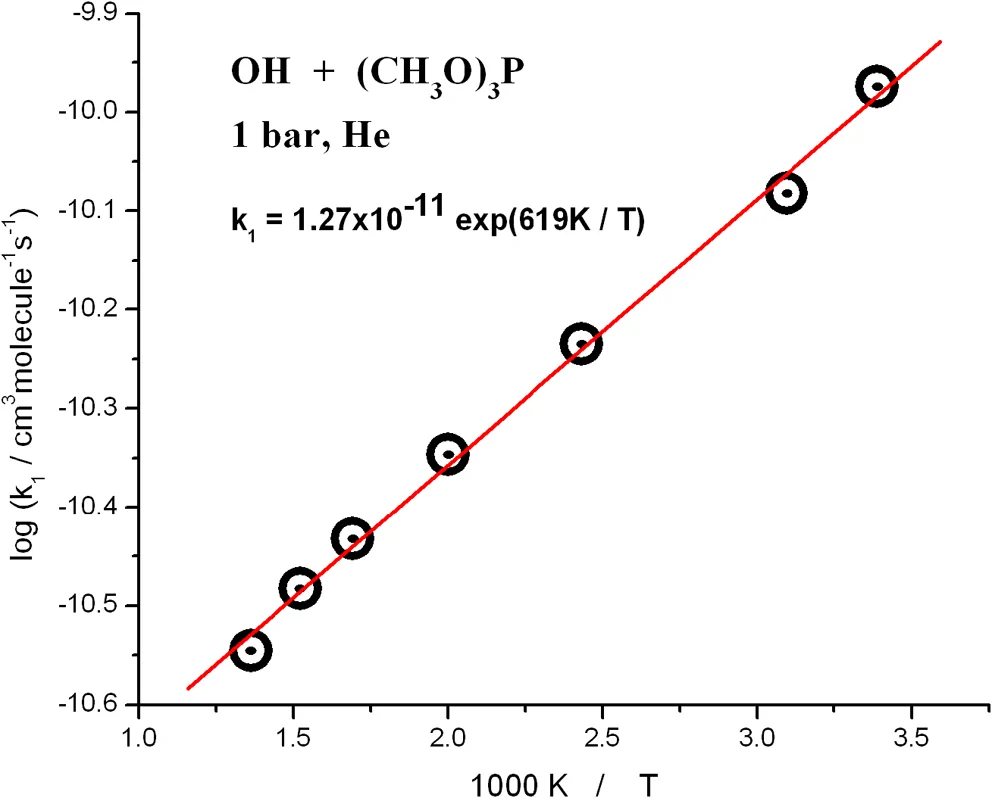
Arrhenius plot showing the apparent activation energy for the reaction of OH with TMPi over the temperature range 295–735 K in 1 bar He.

**Table 1:** Experimental Conditions and Rate Constant of Reaction TMPi + OH at 1 bar, Varied Temperatures

*T*/K	P/bar	[H_2_O]/10^17^	[N_2_O]/10^16^	[TMPi]/10^14^	*k*′/10^3^ s^‑1^	*k*/10^–11^ cm^3^ molecule^–1^ s^–1^
295	1.015	2.5	13.7	3	34.01	10.6
295	1.015	2.5	13.7	2.4	25.77	
295	1.015	2.5	13.7	1.8	17.45	
295	1.015	2.5	13.7	1.2	14.84	
295	1.015	2.5	13.7	0.6	7.87	
295	1.015	2.5	13.7	0	0.62	
323	1.02	1.78	7.27	2.68	23.47	8.28
323	1.02	1.78	7.27	2.14	17.51	
323	1.02	1.78	7.27	1.61	13.12	
323	1.02	1.78	7.27	1.07	8.47	
323	1.02	1.78	7.27	0.54	5.65	
323	1.02	1.78	7.27	0	0.45	
411	1.017	1.8	9.88	2.7	16.34	5.82
411	1.017	1.8	9.88	2.16	12.39	
411	1.017	1.8	9.88	1.62	8.47	
411	1.017	1.8	9.88	1.08	6.67	
411	1.017	1.8	9.88	0.54	3.02	
411	1.017	1.8	9.88	0	0.34	
500	1.028	1.79	8.19	2.24	10.04	4.5
500	1.028	1.79	8.19	1.79	7.75	
500	1.028	1.79	8.19	1.34	5.99	
500	1.028	1.79	8.19	0.89	3.44	
500	1.028	1.79	8.19	0.45	1.43	
500	1.028	1.79	8.19	0	0.24	
591	1.018	1.55	8.53	2.33	8.7	3.7
591	1.018	1.55	8.53	1.86	7.25	
591	1.018	1.55	8.51	1.39	5.24	
591	1.018	1.55	8.51	0.93	3.33	
591	1.018	1.55	8.51	0.46	1.83	
591	1.018	1.55	8.51	0	0.24	
657	1.003	1.37	7.53	3.09	10.45	3.29
657	1.003	1.37	7.53	2.45	7.87	
657	1.003	1.37	7.53	1.85	6.06	
657	1.003	1.37	7.53	1.23	4.37	
657	1.003	1.37	7.53	0.62	1.32	
657	1.003	1.37	7.53	0	0.52	
735	1.009	1.23	6.77	3.7	10.72	2.85
735	1.009	1.23	6.77	2.96	8.55	
735	1.009	1.23	6.77	2.22	6.49	
735	1.009	1.23	6.77	1.48	3.75	
735	1.009	1.23	6.77	0.74	1.93	
735	1.009	1.23	6.77	0	0.54	

As noted above, TMPi reacts with water to form dimethyl hydrogen phosphite (DMHP) and methanol (CH_3_OH). At ambient temperature, both species react with OH much slower than TMPi, with rate constants of 5 × 10^–12^ and 9 × 10^–13^ cm^3^ molecule^–1^ s^–1^, respectively.^24^ Therefore, it could be expected that interference from the reaction of TMPi with water might lead to the decrease of the observed apparent rate constant. Additional experiments performed with slightly modified network of connecting tubing (where flow of helium containing TMPi was mixed with the flow containing H_2_O right before the entrance of the reactor so that the contact time was ultimately minimized) and the internal wall of the reactor was lined with Teflon film confirmed the rate constant of reaction 1 of 1.0 × 10^–10^ cm^3^ molecule ^–1^ s^–1^ at room temperature.

## Summary

The rate constant for reaction 1 was measured over a broad temperature range up to the thermal stability limit of TMPi using pulsed laser photolysis coupled with transient UV absorption. A clear negative temperature dependence was observed. The measured rate constant is approximately seven times lower than the previously reported experimental value obtained by a relative-rate method and about an order of magnitude higher than those reported for two related organophosphorus compounds, trimethyl phosphate (TMP) and dimethyl methylphosphonate (DMMP).

The substantially greater reactivity of TMPi toward OH compared with TMP and DMMP may be attributed to the electronic structure of the phosphorus center as an important determinant of OH reactivity. TMPi contains a trivalent, trigonal-pyramidal P­(III) center with a phosphorus lone pair, whereas TMP and DMMP contain pentavalent P­(V) centers. The approximately order-of-magnitude enhancement in the rate coefficient for TMPi therefore suggests that reduction of the phosphorus center modifies the entrance-channel interaction with OH and facilitates formation of a reactive encounter complex. This interpretation is further supported by the qualitatively different temperature dependence. The OH + TMP and OH + DMMP reactions exhibit V-shaped behavior, with a turnover to positive temperature dependence at elevated temperatures. In contrast, OH + TMPi retains a negative temperature coefficient up to 735 K. The absence of a turnover within the thermal stability range of TMPi suggests that the low-barrier pathway remains kinetically dominant over the entire experimentally accessible temperature range.

Recent theoretical calculations for H atom abstraction from TMPi predict a particularly low barrier for reaction with OH, below 1 kcal mol^–1^,^25^ supporting the existence of a low-barrier reaction pathway. The present experimental observation of a negative apparent activation energy (−1.23 kcal mol^–1^) further suggests that the measured temperature dependence is influenced not only by the intrinsic abstraction barrier but also by the dynamics preceding passage to products. Thus, the combined kinetic evidence indicates that changing the phosphorus center from P­(V) to P­(III) qualitatively alters the OH reaction dynamics, resulting in rapid OH removal and the absence of the high-temperature kinetic crossover observed for the corresponding P­(V) compounds.
